# Out of Sight, out of Mind? Evidence of Human‐Induced Threats in the Elusive *Ziphius cavirostris* of the Mediterranean Sea

**DOI:** 10.1002/ece3.71790

**Published:** 2025-07-11

**Authors:** Manel Gazo, Odei Garcia‐Garin, Sarah Soler‐Ruiz, Laura Fernández‐Suárez, Antonella Arcangeli, Lea David, Morgana Vighi

**Affiliations:** ^1^ Faculty of Biology, Department of Evolutionary Biology, Ecology and Environmental Sciences, and Biodiversity Research Institute (IRBio) Universitat de Barcelona Barcelona Spain; ^2^ Faculty of Sciences, Department of Environmental Sciences, Institute of Aquatic Ecology (IEA) Universitat de Girona Girona Spain; ^3^ ISPRA Rome Italy; ^4^ EcoOcean Institut Montpellier France

## Abstract

Cetaceans in the Mediterranean Sea are increasingly exposed to a suite of anthropogenic threats, including fisheries interactions, vessel strikes, and environmental degradation. While much is known about these impacts on common species, deep‐diving and elusive cetaceans like the beaked whale *Ziphius cavirostris* remain poorly studied due to their offshore distribution and brief surface intervals. This study reports a rare sighting of *Z. cavirostris* in the Western Mediterranean Sea exhibiting a significant dorsal laceration, potentially linked to human activity. The observation was made during a standardized ferry‐based monitoring survey in the Alboran Sea. We contextualize this sighting within broader data on anthropogenic threats to cetaceans, highlighting the underreporting of sub‐lethal injuries in offshore species. This case underscores the importance of opportunistic platforms and long‐term monitoring networks, such as FLT Med Net, in detecting and evaluating human‐induced impacts on rarely observed marine mammals. Our findings advocate for targeted conservation actions and improved mitigation strategies in high‐risk areas of the Mediterranean Sea to protect vulnerable cetacean populations, including beaked whales.

## Graphical abstract

This study documents a rare sighting of a *Ziphius cavirostris* with a severe dorsal injury in the Western Mediterranean likely linked to human activity. The observation, made during a ferry‐based monitoring survey, highlights the value of opportunistic platforms in detecting underreported sub‐lethal injuries and underscores the need for targeted conservation measures in high‐risk areas.

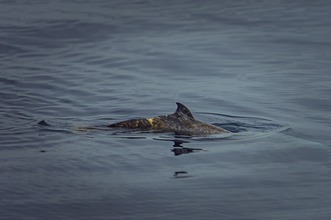

Cetaceans face a wide range of well‐documented, human‐induced threats worldwide, including habitat loss, ecosystem changes, and reduced prey availability driven by climate change and antropization, chemical and acoustic pollution, vessel strikes, as well as bycatch and other fishing‐related interactions (Nicol et al. 2020; Moore 2014; Garcia‐Garin et al. 2021).

Direct (i.e., bycatch) and indirect (i.e., entanglement in or ingestion of discarded gears, etc.) interactions with fisheries currently stand as the most significant anthropogenic threat to cetaceans (Ritchie and Roser 2021). The spatial and temporal overlap between cetaceans and fishing fleets that share the same waters leads to frequent interactions, which have been steadily increasing from the 18th century through the early 2000s and have been widely documented worldwide (Lewison et al. 2014; Snape et al. 2018).

In the Mediterranean, evidence from direct observations and interviews with fishers suggests that by‐catch rates remain generally low (Gonzalvo et al. 2008; Fortuna et al. 2010; Izquierdo‐Serrano et al. 2022), with some differences related to the type of fisheries. Indeed, despite depredation by dolphins on artisanal fisheries being common, often resulting in damaged gear and economic losses of commercial catches, by‐catch in these small‐scale artisanal fisheries remains comparatively low (Lauriano et al. 2004; Díaz López 2006; Gazo et al. 2008). In contrast, pelagic longlining and purse seining have been associated with significantly higher by‐catch rates, particularly affecting small delphinids in the Western Mediterranean (Zahri et al. 2007; Macías‐López et al. 2012).

Vessel strikes (i.e., collisions between vessels and cetaceans or other marine fauna) also pose a significant pressure to cetaceans (Vighi 2025a). With the constant rise of maritime traffic and the parallel introduction of faster and larger vessels, the incidence of this issue has globally increased since the 1950s (Carrillo and Ritter 2010; Laist et al. 2001). While large whales are most often affected by vessel strikes due to their swimming and diving behavior, smaller species may also be at risk, although data on these is limited (Jensen and Silber 2003; Van Waerebeek and Leaper 2008; Winkler et al. 2020). The issue is particularly relevant in regions where maritime traffic is more intense, such as archipelagos (e.g., the Canary Islands, Ritter et al. 2019), busy shipping routes (e.g., the Strait of Gibraltar, Scuderi et al. 2024), and the Mediterranean Sea, one of the most trafficked basins worldwide, which, despite covering less than 1% of the ocean's surface, accounts for nearly 15% of global sea trade (Randone et al. 2019). In a global analysis of vessel strikes across FAO Major Fishing Areas, Winkler et al. (2020) ranked the Mediterranean Sea (FAO Area 37) as second in collision incidents, after the northeastern coast of the United States, Canada, and Greenland's western coast (FAO Area 21).

Being critically affected by human‐induced threats, the need for regulatory measures to mitigate these impacts in the Mediterranean Sea has become increasingly evident (Nisi et al. 2024). In response to this growing concern, the several institutions involved in cetacean conservation (such as the International Whaling Commission—IWC or the Agreement on the Conservation of Cetaceans of the Black Sea, Mediterranean Sea and Contiguous Atlantic Area—ACCOBAMS) have devoted attention to promote the implementation of conservation measures, including the introduction of acoustic deterrent devices (also known as pingers) to keep cetaceans distant from fishing gears, the adoption of modified fishing gear types, the implementation of temporal and spatial closures, speed regulations, re‐routing measures, and watchkeeping arrangements (e.g., IWC 2022; Leaper et al. 2018; Dawson et al. 2013). The definition of marine protected areas (MPAs), particularly sensitive species areas (PSSAs), and important marine mammal areas (IMMAs) also plays a relevant role in the implementation of such measures. Key examples of such areas include the Cetacean Migration Corridor and the Pelagos Sanctuary, which have been recently designated as a PSSA (NW Mediterranean PSSA, Figure 1).

**FIGURE 1:**
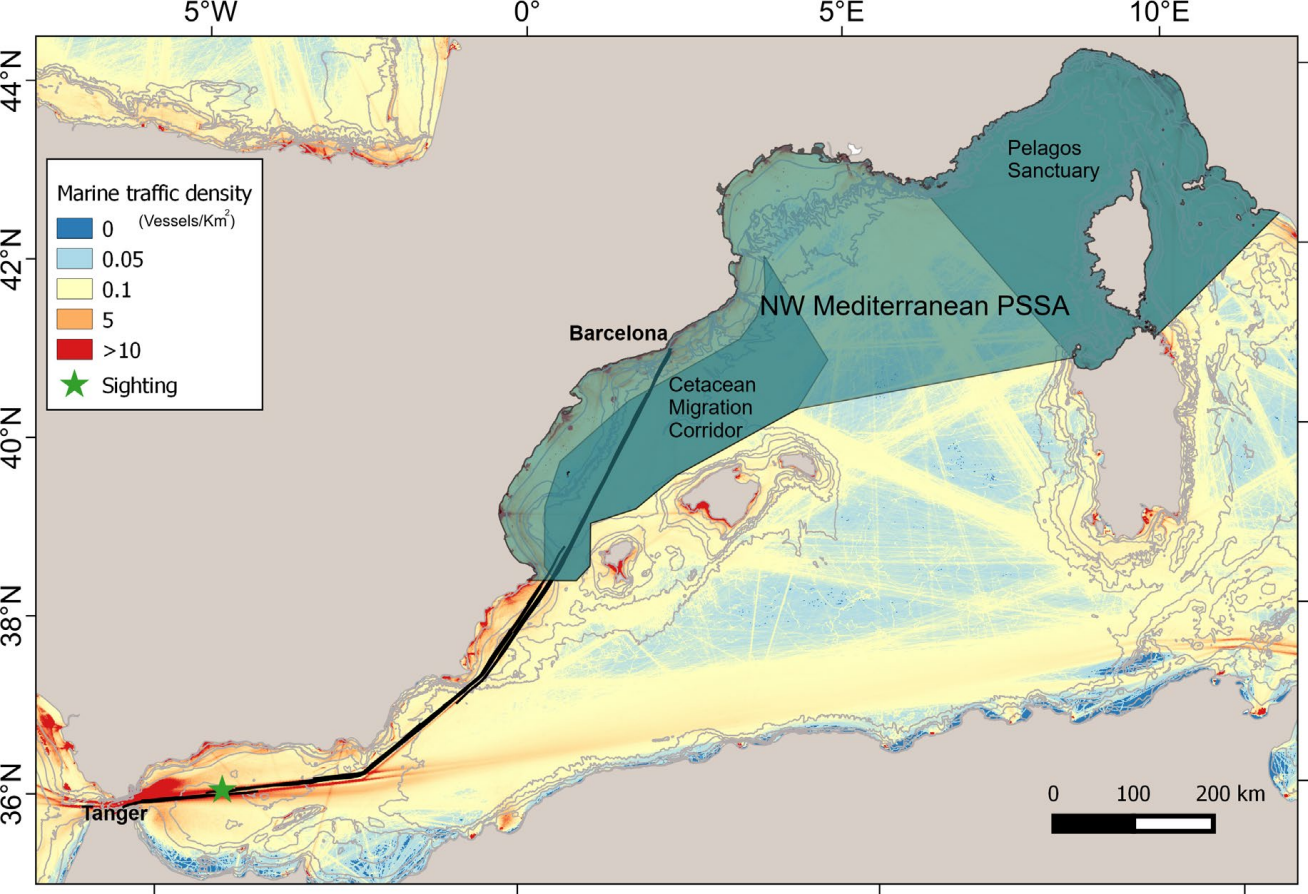
Transect route (black lines) Barcelona‐Tangier‐Barcelona, conducted from 4th to 7th November 2024. The green star indicates the *Ziphius cavirostris* sighting on 5th November 2024. Pale green areas represent particularly sensitive sea areas (PSSAs). Data on marine traffic density were obtained from EMODnet (https://emodnet.ec.europa.eu/geoviewer/).

In this decision‐making scenario, it is highly relevant to define which species are most significantly affected by human‐induced threats and the high‐risk contexts, in order to propose tailored measures for each area and species. The assessment of human impacts on highly mobile marine fauna such as cetaceans is not straightforward. While human‐related injuries, such as those caused by entanglement or other interactions with fishing gears, or collisions with vessels, can be detected and identified at different levels of detail through the observation and necropsy of stranded cetaceans, they are yet difficult to observe and define in live animals at sea (Vighi 2025b). However, long‐term routine monitoring programs provide valuable data to support and integrate the assessment of the incidence of such injuries on stranded cetaceans.

Research on stranded cetaceans in the Cetacean migration corridor and surrounding western Mediterranean waters indicates that fisheries interactions are a leading cause of death. Along the Catalan coast, 27.8% of stranded striped dolphins ( *Stenella coeruleoalba* ) and 60% of stranded bottlenose dolphins ( *Tursiops truncatus* ) showed evidence of fisheries‐related injuries (Cuvertoret‐Sanz et al. 2020). Further south, 26.7% of bottlenose dolphins showed signs of fisheries interaction, while striped dolphins had a lower interaction rate of 6.4%. Overall, 7.8% of all stranded cetaceans in the area bore marks of fisheries‐related incidents (Izquierdo‐Serrano et al. 2022). On the other hand, for what concerns vessel strikes, the 2020 Pelagos Sanctuary data report showed that between 6% and 21% of fin whales and 9.1% of sperm whales sighted in the area exhibited collision‐related scars, including propeller marks and cuts on dorsal fins and flukes (Panigada et al. 2020; Di‐Méglio et al. 2018). Higher‐risk areas have been identified by David et al. (2022) in the central and deeper parts of the northwestern Mediterranean Sea and in some sections of the northern Tyrrhenian Sea.

However, comparable data are lacking for elusive species such as beaked whales. This deep‐diving species, which spends minimal time at the surface and predominantly inhabits offshore waters, is likely underrepresented in collision assessments and fisheries entanglements due to the limited opportunities for its observation and data collection. Knowledge about beaked whales in general is scarce (Li and Rosso 2021), and this is especially true for *Ziphius cavirostris* in the Mediterranean Sea. Most information about this species derives from stranded individuals, which often wash ashore in poor conservation conditions to perform a full necropsy. Without clear evidence, such as severe lacerations or entanglement marks, it is often impossible to determine the cause of death. Reports of stranded *Ziphius cavirostris* affected by human‐induced threads are relatively common in regions like the Canary Islands and parts of the Indo‐Pacific Ocean, where vessel strikes are known to result in fatal outcomes (Carrillo and Ritter 2010; Neilson et al. 2012; Nanayakkara and Herath 2017). However, confirmed cases of such incidents remain rare in the Western Mediterranean Sea.

While data obtained from stranded individuals provide invaluable information on the occurrence of human‐related injuries and mortality, this information is undoubtedly an underestimate of their actual incidence since not all cetacean carcasses wash ashore and several strandings go unreported. Observations at sea of injured individuals or of direct interactions, such as depredation of fishing gears or “near miss events” where cetacean encounters occur within 50 m of a moving vessel's bow (David et al. 2022), are also highly relevant to assess the magnitude of threats related to fisheries and maritime traffic. However, this is not an easy task, particularly with species, categorized as deep divers, that are more difficult to observe.

Opportunistic platforms such as ferries or cargo ships that travel along fixed routes have become invaluable tools for collecting data on cetaceans and on their interactions with human activities. A notable example of this approach is provided by the Fixed Line Transect Mediterranean Network (FLT Med Net), an international network of research institutions, universities, and NGOs coordinated by ISPRA (the Italian Institute for environmental research and protection), which has been conducting systematic ferry‐based surveys since 2007 and currently counts 16 cross‐border transects across the Western Mediterranean Sea (Arcangeli et al. 2019). Within this framework, and under the support of the Medsealitter and LIFE Conceptu Maris EU projects, monitoring activities were implemented on the ferry route between Barcelona (Spain) and Tangier (Morocco) since 2018 and have been regularly conducted until February 2020 and again from May 2023 using the standardized protocol defined within the network.

On November 5, 2024, as part of the routine monitoring aboard the GNV ferry *EXCELLENT* traveling along the Barcelona–Tangier route, observers from the FLT network sighted a group of three *Ziphius cavirostris* approximately 250 m from the vessel at coordinates 36°10′56.51″N, 4°8′14.20″W, at an estimated depth of 2000 m (Figure 1). The sighting lasted approximately 1 min, during which the individuals exhibited no behavioral response to the vessel's proximity. The group was observed milling and moving northwards. A large individual briefly surfaced, revealing a notable dorsal laceration located behind its dorsal fin (Figure 2).

**FIGURE 2:**
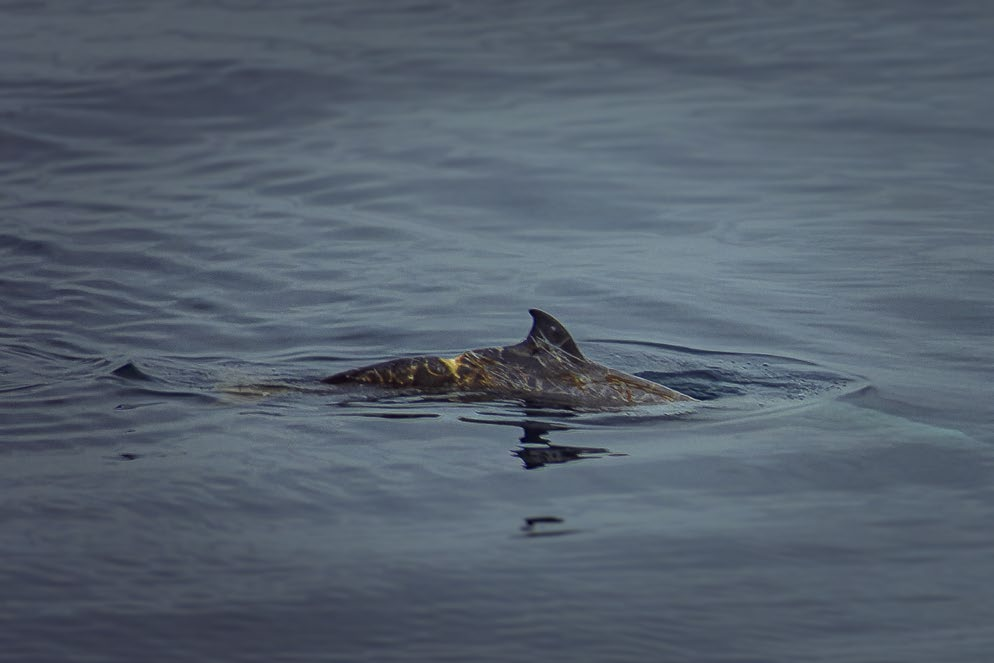
*Ziphius cavirostris* showing a dorsal scar consistent with injuries from a human‐induced interaction. The V‐shaped laceration behind the dorsal fin, partially filled with granulation tissue, suggests a previous entanglement with a fishing gear or contact with a vessel hull. Photo by Sarah Soler‐Ruiz.

The V‐shaped scar was already partially healed with granulation tissue. Since the animal was observed from a distance and it was not possible to collect any tissue, it is difficult to conclusively determine the cause of such injury, and we can only speculate on it. Comparable dorsal scars have been documented in other *Ziphius cavirostris* and are often attributed to natural causes, such as interactions between adult males (Rosso et al. 2011; Gowans and Whitehead 2001). However, the scars reported in the literature referred to as a “back indentation” (Gowans and Whitehead 2001) are generally smaller or less extensive than the one observed here, which appears deeper and more pronounced. Such observation leaves open the possibility that human‐related factors may be responsible, such as entanglement in fishing gear or marine debris, or, to a lesser extent, a collision with the hull of a small vessel. However, when there is no direct evidence of a ship strike, the possibility of an alternative cause should also be considered (Hanninger et al. 2023). Indeed, direct and indirect interactions with fishing operations and entanglement in active or discarded nets, or in floating debris, can lead to wounds and scarring, ultimately increasing stress, injury, and mortality in these species. The hypothesis of a human‐related injury would also be supported by the fact that the beaked whale was sighted in the western Alboran Sea, approximately 60 nautical miles from the Strait of Gibraltar, a region that experiences intense fishing activity, and where interactions between fisheries and cetaceans have been historically documented (Silvani et al. 1999; Tudela et al. 2005; Hanninger et al. 2023). Additionally, the area serves as a major crossroads for large commercial vessels that travel between the Mediterranean Sea and the Atlantic Ocean, making it one of the busiest maritime traffic zones in the Mediterranean (Figure 1) and globally.

This sighting of a *Ziphius cavirostris* bearing non‐lethal human‐related injuries highlights the need to consider multiple anthropogenic threats in conservation assessments for the species in the region. Given that these cetaceans typically inhabit offshore waters, human‐induced injuries and mortality may be underreported, as lethal interactions often go unnoticed when carcasses sink. This emphasizes the importance of recognizing the potential for more widespread impacts on the species in the region than previously understood.

In light of the escalating threats posed by entanglement, vessel strikes, and other human activities, it is crucial to prioritize effective conservation measures and adopt multidisciplinary approaches to protect cetacean populations. The Western Mediterranean Sea is affected by intensive fishing activities, and many fishing gears used in the region have been shown to interact with cetaceans and to pose a significant risk of injury and entanglement for these animals (FAO 2018). While the impact of fishing activities on cetaceans is a well‐documented threat in the area (Hanningher et al. 2023), vessel strikes also remain a major concern that requires further attention, as they often result in injuries and fatalities that are difficult to observe in live animals and diagnose in stranded individuals. The combined efforts of ferry operators that provide free platforms for continuous long‐term monitoring, observers that document the evidence, and researchers that model and analyze these close‐range encounters and overlapping areas are instrumental to complement the information obtained from other sources (Arcangeli et al. 2024; David et al. 2022). Together, they offer invaluable insights into human‐related threats to cetaceans and lay the groundwork for effective mitigation strategies.

## Author Contributions

**Manel Gazo:** conceptualization (lead), data curation (equal), investigation (lead), methodology (equal), resources (equal), writing – original draft (lead), writing – review and editing (equal). **Odei Garcia‐Garin:** conceptualization (equal), data curation (equal), methodology (equal), resources (equal), writing – original draft (equal). **Sarah Soler‐Ruiz:** data curation (equal), investigation (equal). **Laura Fernández‐Suárez:** data curation (equal), investigation (equal). **Antonella Arcangeli:** data curation (equal), methodology (equal), resources (equal), writing – review and editing (equal). **Lea David:** data curation (equal), methodology (equal), resources (equal), writing – review and editing (equal). **Morgana Vighi:** conceptualization (equal), data curation (equal), methodology (equal), resources (equal), writing – original draft (equal).

## Conflicts of Interest

The authors declare no conflicts of interest.

## Data Availability

Data sharing not applicable to this article as no datasets were generated or analyzed during the current study.
